# Oxidant-Antioxidant balance in patients with coronary slow flow

**DOI:** 10.12669/pjms.35.3.162

**Published:** 2019

**Authors:** Sadettin Selcuk Baysal, Sahbender Koc

**Affiliations:** 1 *Sadettin Selcuk Baysal, Department of Cardiology, Sanliurfa Mehmet Akif Inan Training and Research Hospital, 63300, Sanliurfa, Turkey*; 2 *Sahbender Koc, Cardiology Department, Kecioren Training and Research Hospital, 06300, Ankara, Turkey*

**Keywords:** Antioxidant, Biomarker, Coronary slow flow, Oxidant, Oxidative stress

## Abstract

**Objective:**

Recent studies have focused on the probable role of oxidative stress in cardiovascular diseases. We aimed to assess the oxidant/antioxidant biomarkers in coronary slow flow (CSF).

**Methods:**

The study included 51 subjects with CSF and age and sex matched 32 controls. Detailed anamnesis of the patients in the study was taken and routine physical examinations were performed. Routine biochemical blood tests were analyzed. Total oxidative status (TOS), oxidative stress index (OSI) and lipid hydroxyperoxide (LOOH) levels as oxidant biomarkers; paraoxonase (PON1), ceruloplasmin (CP), free sulphydryl (SH) groups, and total antioxidant capacity (TAS) levels as antioxidant biomarkers were studied.

**Results:**

Baseline demographic characteristics of the study population did not differ significantly between groups.TOS, OSI and LOOH concentrations were higher in study group than in control group. However, there was no significant difference detected in levels of TAS, PON1, SH and CP. Multivariate logistic regression analysis revealed that TOS, hsCRP and smoking were indepedent risk factors of CSF.

**Conclusions:**

Although there was not any significant difference in antioxidant biomarkers (TAS, PON1, SH and CP) in CSF patients, we detected increased TOS, OSI and LOOH levels which have oxidant properties. These data supported the possible involvement of oxidative stress in pathogenesis of CSF as previous studies reported.

## INTRODUCTION

Coronary slow flow (CSF) phenomenon is the slow movement of contrast to distal vascular structures during coronary angiography in patients with normal or near normal epicardial coronary arteries.^1^ Although its mortality is not very high, it has a high morbidity due to recurrent chest pain and different ischemic presentations. Endothelial dysfunction, vasomotor dysfunction, microvascular disease and oxidative stress are suggested to be associated with this clinical entity, although the etiopathogenesis still remains unclear.^2^

Free radicals are molecules that contain at least one unconjugated electron in their outer orbit, and are prone to react with other molecules to pair the unstable and single electron. Free radicals make pathological changes in cell membranes, cell organelles and DNA by taking part in oxidation of protein, lipid, carbohydrate and DNA. Particles that prevent or delay the effects of the molecules that may lead the oxidation of structural particles in the organism are defined as antioxidants. The imbalance between oxidants and antioxidants causes extremely formation of reactive oxygen species and oxidative damage. This is referred to as oxidative stress.^3^,^4^ Oxidative stress is thought to take part in the etiopathogenesis of many systemic diseases, including cardiovascular diseases.^5^,^6^

Limited studies have investigated the association between slow flow and oxidative stress so far.^7^-^9^ In this study in patients with CSF, we intended to evaluate the concentrations of total oxidative status (TOS), oxidative stress index (OSI) and lipid hydroxyperoxide (LOOH) which have antioxidant properties; paraoxonase (PON1), ceruloplasmin (CP), free sulphydryl (SH) groups, and total antioxidant capacity (TAS) levels which have oxidant properties.

## METHODS

Between January 2016 and May 2017, 10285 patients who presented to our clinic with stable angina or unstable angina pectoris (USAP) complaints underwent coronary angiography. Of these patients, 51 patients diagnosed with CSF who had no significant lesion in the left main coronary artery, the other three major coronary arteries, and their lateral branches above 2.0 mm and 32 patients with normal coronary arteries and similar age and sex distribution served as a control group were included in our study. Patients who had acute coronary syndromes, coronary ectasia (dilatation of the coronary artery diameter more than 1,5 or more compared to the nearest normal segment) or anomaly, coronary vasospasm, left ventricular dysfunction (ejection fraction ‹ 50%), significant valvular disease, malignancy, acute or chronic inflammatory conditions, chronic obstructive lung disease; liver, kidney thyroid and hematologic disorders were excluded. Detailed anamnesis of the patients in the study was taken and routine physical examinations were performed. Routine biochemical blood tests were analyzed. Hypertension was accepted as the presence of blood pressure above 140/90 mmHg or antihypertensive treatment. Fasting blood glucose above 126 mg / dL; use of oral anti-diabetic medicines or insulin was defined as diabetes mellitus. The study which was approved by local ethics committee (Ref. 74059997.050.01.04/107). , was carried out in accordance with the ethics principles of Helsinki Declaration. Informed consent forms were signed voluntarily after all the study principles were explained to all patients.

Coronary angiographies were performed on the Siemens Axiom Sensis XP angiography device using Judkins technique in a femoral or radial percutaneous approach. Coronary arteries were visualized at 30 frames per second (30 fps) with cranial and caudal angles in left and right oblique planes. Iopromide (Ultravist-370) was used as contrast agent. Two cardiologists who were blinded to the study reviewed the angiograms and evaluated the coronary blood flow rates by using the thrombolysis in myocardial infarction (TIMI) frame count method (TFC) as previously described.^10^ The intra- and inter-observer coefficients of variation were 4.3 and 6.1, respectively. The mean TIMI frame count was calculated as the average of the RCA, Cx and LAD TIMI frame counts. In case of any coronary artery with slow flow was seen enough to randomize patients in CSF group.

Blood samples for evaluating oxidant-antioxidant parameters were obtained by taking 10 cc of blood from the sheath prior to withdrawal of the sheath of patients who were underwent routine biochemical and haematological evaluation before coronary angiography. They were centrifuged for five minutes at 5000 rpm and divided into serum. Then the serums were placed in plain tubes and stored at -80°C.

The serum TAC and TOS levels were determined with a novel automatic method, developed by Erel.^11^,^12^ The ratio of TAC to TOS is defined as OSI, expressed as percentage. The measurement of CP was performed by Erel method^13^ which is an automatic and calorimetric method based on enzymatic oxidation of ferric ion to ferric ion. Serum LOOH levels were evaluated in the acidic medium with ferric ion oxidation-xylenol orange (FOX-2) method based on enzymatic oxidation of ferric ion to ferric ion by lipid hydroperoxides.^14^ Serum SH levels were determined according to Ellman and Hu methods^15^,^16^, as described previously. Basal serum PON1 activity was measured spectrophotometrically in the absence of sodium chloride. It was measured using paraoxon as a substrate and measuring the absorbance of 37°C at 412 nm in the color formed by the hydrolysis of paraoxone.^17^

### Statistical analysis

All statistical analyses were performed by SPSS for Windows software (ver.22.0; SPSS Inc., Chicago, IL, USA). The distributions of continuous variables were evaluated by the Shapiro-Wilks test. For comparison of continuous variables, Independent samples t-test (parametric) or Mann –Whitney U Test (non-parametric) were applied whenever appropriate. Descriptive statistics were expressed as mean and standard deviation for normally distributed variables, median and minimum- maximum for non normally distributed ones. Spearman and Pearson correlation coefficients were assessed due to distributions of the data. Logistic regression analyses were performed to find independent risk factors for CSF. Variables with p<0,25 in the univariate analyses were included to the multivariate model. Sensitivity, specificity and cut-off values of each biomarker were determined by receiver operating characteristic (ROC) analysis. ROC curves are shown for biomarkers with a statistically significant difference between groups. A p-value < 0.05 was considered to indicate statistical significance.

## RESULTS

A total of 83 patients were enrolled in the study, 51 of them (61.4%) are CSF cases and 32 of them (38.6%) are controls. Baseline features of study population are represented in Table-I. Clinical and laboratovary data of case and control groups were similar except high-sensitivity C-reactive protein (hsCRP) (p<0,05). HsCRP levels were significantly increased in CSF group compared to control group (0.68±0.09 vs. 0.41±0.05, p<0.001).

**Table-I:** Clinical, laboratory and angiographic data of the study population.

	SCF (n=51)	NCF (n=32)	p
***Clinical data***			
Age, years	54±8	53±7	0.911
Male, n (%)	36 (70)	20 (62)	0.334
BMI, kg/m²	27.4±4.3	26.9±3.5	0.584
Diabetes mellitus, n (%)	15 (29)	10 (31)	0.814
Hypertension, n (%)	22 (43)	12 (37)	0.546
Smoking, n (%)	22 (43)	9 (28)	0.051
***Laboratory data***			
Total cholesterol, mg/dl	218.5±41.4	199.5±37.3	0.203
LDL, mg/dl	117.5±27.7	104.1±27.4	0.221
HDL, mg/dl	32.1±8.8	34.3±6.4	0.733
Triglyceride, mg/dl	234±65	203±48	0.486
Fasting glucose, mg/dl	104.3±21.4	100.4±21.2	0.384
Creatinine, mg/dl	0.76±0.11	0.74±0.12	0.399
Hemoglobin, g/dl	15.7±1.3	15.1±1.2	0.557
WBC, 10³/mm³	8.17±1.15	8.51±1.58	0.481
Neutrophil, 10³/mm³	4.98±1.13	4.76±0.82	0.355
Lymphocyte, 10³/mm³	2.99±0.54	3.27±0.62	0.117
Platelet, 10³/mm³	218.6±38.3	231.4±31.6	0.321
hsCRP, mg/dl	0.68±0.09	0.41±0.05	<0.001
***TIMI frame count measurements***			
LAD (corrected)	35.2±6.3	21.2±1.1	<0.001
Cx	31.5±7.2	18.4±1.2	<0.001
RCA	30.7±8.2	18.3±1.8	<0.001
Mean	31.9±7.3	19.4±1.5	<0.001

BMI: Body Mass Index, Cx: Circumflex artery, HDL: High-Density Lipoprotein,

hsCRP: High Sensitive C reactive Protein, LAD: Left Anterior Descending Artery,

LDL: Low-Density Lipoprotein, RCA: Right Coronary Artery, WBC: White Blood Cell.

The results of oxidative and antioxidative biochemical analysis of the patients in the study are shown in Table-II. TOS, OSI and LOOH concentrations that reflect oxidative stress were significantly increased in CSF group. On the other hand, the values of biomarkers considered to have a role in antioxidant system such as TAS, PON1, SH and CP were similar between the groups (p<0.05).

**Table-II:** Oxidative/ anti-oxidative biomarker levels of the study population.

	SCF (n=51)	NCF (n=32)	p
TOS(mmol H2O2 equiv./L)	31.45[17.09-51.84]	24.35[17.88-40.28]	0.005*
TAS(mmol Trolox equiv /L)	1.03±0.21	1.02±0.18	0.834**
OSI(arbitrary unit)	3.23±1.23	2.54±0.56	0.001**
PON1(U/L)	74.57[39.98-110.42]	76.33[44.6-108.5]	0.735*
LOOH (μmol H2O2 Eqv./L)	11.67[8.27-22.64]	10.33[8.77-21.47]	0.02*
SH (mmol/L)	0.37±0.07	0.40±0.06	0.138**
CP (U/L)	427.11±85.00	445.19±96.92	0.382**

CP: Ceruloplasmin, LOOH: Lipid Hydroperoxide, OSI: Oxidative Stress Index, PON1: Paraoxonase 1, SH: Sulphydryl Groups, TAS: Total Antioxidant Status, TOS: Total Oxidative Status,

** :Independent samples t-test,

* :Mann-Whitney U Test

**Fig.1:**
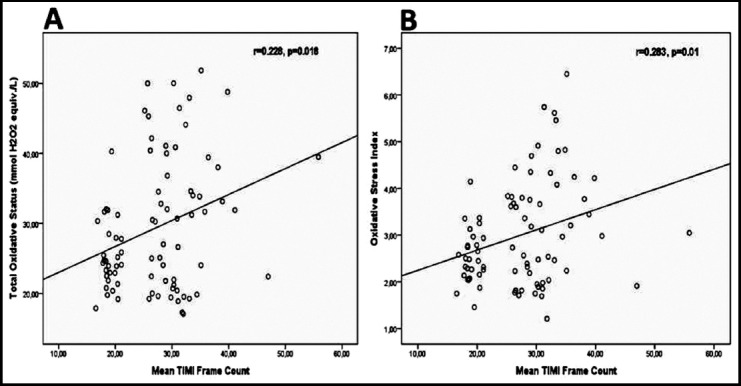
Scatter plots of correlations of total oxidative status (A) and oxidative stress index (B) biomarkers with mean TIMI frame count

In correlation analysis, TOS and OSI concentrations showed a significantly positive correlation with mean variable(p<0.05). Scatter plots were used to show the association for these pair of variables. Table-III reveals the correlation analysis results while Fig.1 shows the scatterplot of these variables.

**Table-III:** Correlation coefficients of mean TIMI frame count to study biomarkers.

	Correlation Coefficients (r)**	p
TOS	0.228	0.018
TAS	-0.118	0.286
OSI	0.283	0.010
PON1	-0.026	0.814
LOOH	0.214	0.052
SH	-0.167	0.131
CP	-0.167	0.132

CP: Ceruloplasmin, LOOH: Lipid Hydroperoxide, OSI: Oxidative Stress Index, PON1: Paraoxonase 1, SH: Sulphydryl Groups, TAS: Total Antioxidant Status, TOS: Total Oxidative Status,

** Spearman Correlation Coefficient was used for all biomarkers, except SH. Pearson correlation coefficient is used for this biomarker.

Multivariable logistic regression analysis was used to detect the major risk factors for SCF. HsCRP, platelet, lymphocyte, BMI and smoking variables were analyzed in logistic regression analysis along with oxidative/anti-oxidative biomarkers. All risk factors and these biomarkers were included to univariate logistic regression analysis. In multivariable logistic regression analysis, Backward: Wald was used as variable selection method. Only TOS was analyzed along with smoke and hsCRP risk factors. As a result, hsCRP and smoking variables were found to be the major risk factors for SCF. (odds ratio for hsCRP: 13.903, 95% confidence interval [CI]: 2.590-74.628, p < 0.05; odds ratio for smoking: 3.76, 95% confidence interval [CI]: 1.104-12.8, p < 0.05). TOS was also found to be risk factor for SCF.(odds ratio for totoxi: 1.095, 95% confidence interval [CI]: 1.016-1.181, p < 0.05). Table-IV shows the logistic regression analysis results.

**Table-IV:** Univariate and Multivariate Logistic Regression Analysis Results.

	Univariate		Multivariate	

	OR (95% CI)	p	OR (95% CI)	p
TAS	1.813(1.174-2.799)	0.007		
PAN1	1.007(1.002-1.013)	0.011		
LOOH	1.062(1.023-1.103)	0.002		
TOS	1.027(1.011-1.043)	0.001	1.095(1.016-1.181)	0.017
CP	1.001(1.000-1.002)	0.013		
SH	1.014(1.003-1.026)	0.016		
OSI	1.297(1,113-1,510)	0.001		
HsCRP	1,546(1,193-2,005)	0.001	13.903(2.590-74.628)	0.002
Smoking	3.385(1.24-9.239)	0.017	3.76(1.104-12.8)	0.034
Platelet	1.021(1.003-1.039)	0.042		
Lymphocyte	1.015(1.000-1.030)	0.046		
BMI	1.029(1.007-1.039)	0.005		

BMI: Body Mass Index, CP: Ceruloplasmin, hsCRP: High Sensitive C reactive Protein,

LOOH: Lipid Hydroperoxide, OSI: Oxidative Stress Index, PON1: Paraoxonase 1,

SH: Sulphydryl Groups, TAS: Total Antioxidant Status, TOS: Total Oxidative Status

**Fig.2:**
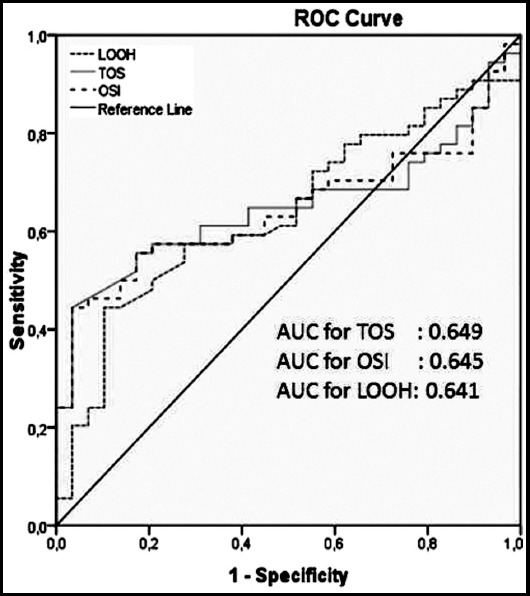
Receiver operating characteristic (ROC) curve for total oxidative status (TOS), oxidative stress index (OSI) and lipid hydroxyperoxide (LOOH) levels to detect CSF. AUC, area under the curve.

ROC Curve Analysis was performed to determine the discriminatory capacity of TOS, OSI and LOOH levels. Area Under Curve value is 0.649 for TOS (95% CI: 0.533-0.766; p<0.05). With the cut-off value of 26.24; sensitivity and specificity values were 61.9% and 66.9%, respectively. Area Under Curve value is 0.645 for OSI (95% CI: 0.528-0.762; p <0.05). With the cut-off value of 2.69; sensitivity and specificity values were 61.1% and 63.1%, respectively. Area Under Curve value is 0.641 for LOOH (95% CI: 0.517-0.757; p<0.05). With the cut-off value of 10.36; sensitivity and specificity values were 61.1% and 61.7%, respectively. Fig.2 shows the ROC curves for these biomarkers.

## DISCUSSION

We found higher oxidant related biomarkers TOS, OSI and LOOH concentrations in CSF patients than in normal population. Another finding of our study is that TOS, hsCRP and smoking were independently associated with CSF. However, there were no differences between groups in the levels of TAS, PON1, SH and CP known as antioxidant.

CSF is an entity characterized by the late arrival of contrast agent to the end of the coronary arteries without atherosclerotic lesions. It was detected in 1-4% of patients undergoing coronary angiography. Although the rate of hospitalization and cardiac catheterization is high in patients with CSF, the prognosis is known to be similar to the normal population.^2^ Although there are many studies on CSF, the underlying mechanism, diagnostic criteria’s, treatment, follow-up and clinical importance have not been fully clarified. Various mechanisms such as endothelial dysfunction, subclinical atherosclerosis, microvascular disease, vasomotor dysfunction, inflammation and oxidative stress have been considered to have a role in the etiopathogenesis. In addition, some studies have shown that this condition is a systemic vascular abnormality rather than a local disorder.^18^,^19^

Studies have demonstrated the role of oxidative stress in the development and progression of many important heart diseases such as endothelial dysfunction, atherosclerosis, hypertension and heart failure.^5^,^20^ Also, the relationship between oxidative stress and CSF has been examined by previous studies. Enli et al.^7^ concluded that free radical damage could play a role in the pathogenesis of CSF by finding reduced levels of glutathione and increased serum malondialdehyde and erythrocyte superoxide dismutase levels in CSF patients. Yucel et al^8^ found higher concentrations of TOS and OSI in SCF patients. In their report plasma TOS concentrations were independently associated with mean TIMI. Kundi et al.^9^ reported that thiol disulfide homeostasis, which is an indicator of oxidative stress, was associated with CSF. Similarly, we found that TOS and OSI levels increased in CSF patients. LOOH is a toxic by-product that is produced by lipid peroxidation caused by the reaction of lipids on the cell membrane with free radicals.^14^ Yildiz et al.^21^ detected that LOOH levels with oxidant properties were similar to the normal population in patients with CSF. On the contrary our study presented higher LOOH levels in CSF subjects. All these findings support the hypothesis that oxidative stress has a role in the pathogenesis of CSF. However, it is not evident whether oxidative stress is one of the causes of this phenomenon or is the result of metabolic processes related to this condition.

PON1 is an antioxidant enzyme in the structure of HDL cholesterol that has the effect of preventing lipoprotein oxidation by hydrolyzing lipid peroxides in oxidized LDL (oxLDL) structure. Previous studies have reported lower serum PON1 activity in atherosclerotic diseases, myocardial infarction, hypercholesterolemia and diabetes mellitus.^22^ Yildiz et al.^21^ also demonstrated lower PON1 activity in CSF subjects than in controls. Contrarily there was not any difference in measured PON1 activity between groups in our study.

CP is a serum protein belonging to the α2-globulin family. It is a protein with antioxidant and acute phase reactant that has function in transporting copper in plasma (95%), oxidation of organic amines, ferrooxidase activity, and regulation of cell iron levels. CP exhibits function as antioxidant by taking part in ferroxidase activity, ascorbate oxidase activity, oxygen radical clearance activity and GSH dependent peroxidase activity.^23^,^24^ Serum SH groups function as important cellular cleaners of peroxides and prevent the cells from being damaged by these molecules. Thus, it is considered that SH groups have a protective effect against oxidative stress.^25^ We measured the levels of these two antioxidant markers in our work and we did not see any significant difference between the groups.

In the light of all the data we obtained in our study, high levels of TOS, OSI and LOOH in SCF patients indicated that oxidant load increased in this patient group. On the other hand, no significant change in the antioxidant properties of TAS, PON1, CP and SH levels in CSF subjects compared to the controls showed that the oxidant load did not trigger the antioxidative mechanisms sufficiently. Based on all these findings, we can speculate that the oxidant/antioxidant balance in patients with CSF is impaired in the oxidant direction.

We find this work worth reporting for two reasons. First, we found that the CP levels of our study were the first to be evaluated in CSF patients according to our literature review. Secondly, it is important to confirm the role of oxidative stress in the pathophysiology of SCF with such a wide biochemistry panel including 7 biomarkers in accordance with previous studies.

### Limitation of the study

It is the small number of subjects. This may be the reason of our inability to detect a significant change in antioxidant biomarker levels. Cross-sectional design is another limitation of our study to understand exact mechanism of CSF. Lastly, we did not use intravascular ultrasound or optical coherence tomography to detect normal coronary artery subjects, our diagnosis depended on the visible angiograms which could lead to bias in randomization of study population.

## CONCLUSION

According to our analysis, oxidant biomarkers (TOS, OSI and LOOH) were higher in CSF group than controls. However, no significant change was observed in antioxidative parameters (TAS, PON1, SH and CP). These findings suggest that oxidative stress in the direction of oxidation could contribute to the pathogenesis of CSF or could be the result of the pathological pathways causing CSF. Further larger investigations are needed to reveal the precise function of oxidative stress in the pathophysiology of CSF.
